# The Role of ApoE Polymorphism in the Relationship between Serum Steroid Hormone Levels and Cognition in Older Chinese Adults: A Cross-Sectional Study

**DOI:** 10.3389/fendo.2018.00071

**Published:** 2018-03-06

**Authors:** Xiaochen Huang, Shengqi Dong, Jie Zhen, Huiqiang Zhang, Tong Lin, Yuhong Zeng, Nicholas Van Halm-Lutterodt, Linhong Yuan

**Affiliations:** ^1^School of Public Health, Capital Medical University, Beijing, China; ^2^Department of Neurosurgery, Beijing Tiantan Hospital, Capital Medical University, Beijing, China; ^3^Keck Medical Center of USC, Department of Orthopaedics and Neurosurgery, University of Southern California, Los Angeles, CA, United States

**Keywords:** apolipoprotein E, polymorphism, steroid hormone, cognition, geriatrics

## Abstract

**Background:**

Epidemiology studies have indicated an association of apolipoprotein E (ApoE) genetic polymorphism and circulating steroid hormone levels with the risk of Alzheimer’s disease. The established physiologic relationship between apolipoproteins and steroid hormone indicate an important role of ApoE polymorphism in impacting the relationship between serum steroid hormones and cognition in the elderly.

**Study design:**

A total of 500 Chinese adults aged between 50 and 75 participated in this community-based cross-sectional study. Blood samples were collected in the morning for ApoE genotyping and serum parameter assessment. Cognitive performance of participants was evaluated by Montreal Cognitive Assessment test.

**Results:**

Age, gender, educational level, smoking, and physical activity levels are factors associated with cognitive performance in this older Chinese adults. Compared to the control subjects, MCI subjects demonstrated higher serum total cholesterol, HDL-C, and estradiol status (*P* < 0.05). ApoE genotype difference of serum lipid profile was observed with a relatively higher mean serum triglyceride levels in ApoE2 and ApoE4 carriers (*P* < 0.05), and lower mean serum HDL-C level in ApoE4 carriers (*P* < 0.05). Memory and delayed recall ability was serum estradiol level related; and subjects with higher circulating estradiol concentration exhibited lower memory and delayed recall ability (*P* < 0.05). The association of serum estradiol and cortisol concentration with cognitive performance was ApoE genotypes dependent. Poor cognitive performance was observed in ApoE2 and ApoE4 carriers with higher serum estradiol level (*P* < 0.05). Moreover, ApoE2 and ApoE4 carriers with higher serum cortisol status demonstrated decreased language ability (*P* < 0.05). Multiple logistic regression analysis indicates that subjects with higher serum estradiol status may have an increased risk for MCI [OR = 2.004, 95% confidence interval (CI): 1.135, 3.540; *P* = 0.017]. ApoE2 carriers with higher serum steroid levels may be potentially predisposed to an increased risk of MCI (OR = 3.353; 95% CI: 1.135, 9.907; *P* = 0.029).

**Conclusion:**

Cognitive outcomes in older Chinese adults are associated with serum steroid hormone status. Higher serum steroid levels in ApoE2 carriers might pose an increased risk of MCI in the elderly.

## Introduction

Due to the prolongation of human life expectancy and the increase of elderly population in China, the prevalence of dementia is logically expected to rise. Alzheimer’s disease (AD) is the most common dementia in the elderly. Until now, the cause of AD remains entirely unclear. Increasing evidence suggests that AD stems from a combination of environmental and hereditary factors (1). Nevertheless, implemented efforts to identify environmental and genetic risk factors associated with AD have not been satisfactory. Thus, the predictor identification of cognitive decline may help to forge and test for strategies in preventing dementia (2).

Circulating steroid hormones are generated from metabolism of adrenal and gonadal steroids (3). A disorder of the hypothalamic–pituitary–adrenal (HPA) axis has been linked to cognitive decline and dementia (4–6). *In vivo* cortisol levels have also been proven to influence explicit memory in humans and other primates (7). However, a prolonged exposure to cortisol over a lifespan period was found to play a significant role in aging process, and potentially contributing to the pathogenesis of mild cognitive decline (8, 9). Interestingly, with advancement in age, sex steroid levels tend to gradually decrease and this decline in steroids may be important for the reported incidence of cognitive decline in the elderly (10–12). The protective roles of sex steroid hormone in AD have been proven by population-based epidemiology studies, also by experimental animal models (13, 14). Significant gender differences of AD exist, with women being at higher risk of AD than men (15). Female AD patients tend to pertain a relatively lower cerebral estrogen levels compared to age-matched cognitively normal controls (16, 17). The decline in estrogen status in postmenopausal women evidently predisposes to accelerated precipitation of AD-like pathology in Alzheimer transgenic model (18, 19).

Both human- and animal-based studies demonstrate significant correlations between *in vivo* steroids and risk of AD. Currently, few studies have explored the role of *in vivo* steroids in affecting cognitive performance in non-demented elderly Chinese population. Additionally, conflicting results regarding the relationship between circulating steroids and dementia have been reported (20). Hence, the relationship between *in vivo* steroids status and cognitive performance is worth considering and complementing insights into the role of endocrine factors and their impacts in influencing the development of cognitive changes.

Gene polymorphisms have been demonstrated to enhance the MCI and AD susceptibility (21). Apolipoprotein E (ApoE) is the best documented genetic risk factor for AD; especially ApoE ε4 allele has been extensively reported as a major risk factor for sporadic AD (22). Due to the fact that steroid hormone is formed from a primary source of cholesterol; physiologic relationship has been established between apolipoproteins and steroids (23). Current literature evidence indicates a possible interaction between steroid hormones and ApoE allelic types in modifying the susceptibility to AD (24, 25). Association between ApoE genetic polymorphism and basal cortisol levels in healthy older adults have also been reported (26). Additionally, the neuroprotective effects of estrogen might be modulated by ApoE genotypes. To date, the relationship between ApoE genetic polymorphism and steroids and their combined effects on cognitive performance is seldom reported in Chinese population. Given the previously reported association between ApoE and cognitive function in the elderly (24, 25), we hypothesize that ApoE gene polymorphism might interact with steroid hormones in affecting cognitive performance in non-dementia elderly Chinese. The purpose of the present study is to explore the association between *in vivo* estrogen and cortisol status with cognitive function in subjects with different ApoE genotypes.

## Materials and Methods

### Participants

A total of 500 participants aged 50–75 (mean age: 62.63 ± 6.16 years) were randomly recruited from Nanyuan Community (Beijing, China). As per our previous published documents (27), the subjects with severe diseases or conditions known to affect cognitive function (e.g., inflammatory diseases, recent history of heart or respiratory failure, chronic liver disease or renal failure, malignant tumors, a recent history of alcohol abuse, history of cerebral apoplexy, or cerebral infarction) were excluded from the study. Moreover, subjects with clinically diagnosed dementia and Parkinson’s disease, long-term frequency intake of medication and antidepressants acting on central nervous system, or those using hormone therapies were also excluded. The current study was approved by the Medical Ethics Committee of Capital Medical University (no. 2012SY23) and written informed consents were obtained from all participants.

### Anthropometric Measurements and Sociodemographic Variables

The anthropometric parameters measured in the present study included height and weight of each participant. Body mass indices (BMIs) were calculated as weight (kg)/height (m)^2^. Self-administered questionnaires adopted from our previous studies (28) was used for the collection of demographic characteristics including age, gender, nationality, education, lifestyle factors [e.g., living condition (living alone, yes or no), smoking (yes or no), alcohol drinking (yes or no), physical activity (never, 1–3 times/week, 4–5 times/week, everyday), reading habit (yes or no), and housekeeping (yes or no)], AD family history (yes or no), medical history of chronic diseases, and dietary supplements. Education level was classified into six categories (illiterate, primary school, junior high school, high school, junior college, undergraduate, and above) based on the highest level attained.

### Blood Measurement

#### Measurement of Serum Parameters

Fasting venous blood samples was collected from each subject in the morning (between 8 a.m. and 9 a.m.). Then, the blood samples were centrifuged in vacuum tubes at 480 *g* for 10 min at 4°C, serum was separated within 2 h, and then stored at −80°C before further analyses. Serum parameters including glucose (Glu), triglyceride (TG), and total cholesterol (TC) were measured by an ILAB8600 clinical chemistry analyzer (Instrumentation Laboratory Lexington, WI, USA). High-density lipoprotein cholesterol (HDL-C) was determined by using a commercially available assay from Instrumentation Laboratory (Lexington, WI, USA). Low-density lipoprotein cholesterol (LDL-C) concentration was calculated according to the method described by Friedewald formula (29). All samples of each subject were analyzed within a single batch, and the interassay coefficients of variation (CV) for all determinations were less than 5%.

#### Measurement of Serum Estrogen and Cortisol

Serum estradiol was determined utilizing a radio-immunassay kit according to the method described previously (30). The quoted CV was less than 10% (kit information). A competitive electrochemiluminescence immunoassay (Elecsys Cortisol; Roche Diagnostics GmbH, Mannheim, Germany) was used for cortisol concentration measurement with a total CV less than 5%.

#### DNA Isolation and Genotyping

DNA was extracted from 2 ml intravenous peripheral blood (stored in −80°C) by using a Wizare genomic DNA purification kit (Promega, Madison, WI, USA). Polymerase chain reaction amplification and restricted fragment length polymorphism analysis were applied for ApoE genotyping according to the method described by Hixson (31). The specific primers used for ApoE genotyping are forward, 5′-GGC ACG GCT GTCCAA GGA-3′; reverse, 5′-GCC CCG GCC TGG TAC ACT GCC-3′. 20% of DNA samples were genotyped again by different operators for the purpose of quality control. For ApoE genotypes, subjects with the E2/E2 and E2/E3 genotypes were grouped as E2 carrier; subjects with E3/E3 were classified as E3 homozygote; and subjects with E3/E4 or E4/E4 were grouped as E4 carrier.

### Cognitive Tests

The cognitive assessment was performed in the morning immediately after the collection of blood sample. Global cognitive function was achieved using the Montreal Cognitive Assessment (MoCA) by recruited and well trained physicians within the community health service center. As a cognitive screening tool, MoCA test was extensively applied for early detection of MCI and AD (32, 33), which consisted of seven cognitive domains including visual-spatial and executive ability, naming, attention, abstraction, language, delayed memory recall and orientation functions. The cutoff points used for MCI diagnosis in the present study were as follows: 13/14 for individuals with no formal education, 19/20 for individuals with 1–6 years of education, and 24/25 for individuals with 7 or more years of education. This cutoffs were sensitive and efficient in the diagnosis of MCI in elderly Chinese (34).

### Statistical Analyses

Data were analyzed with SPSS version 19.0 (Chicago, IL, USA). Continuous variables were presented as mean ± SD or mean [95% confidence interval (CI)]. Gender, smoking, alcohol drinking, and physical activity were presented as category variables. Descriptive statistics were carried out on the data to investigate differences between MCI subjects and controls. General linear model (GLM) was used to compare the means of the detected parameters between the groups. Confounding factors included in the analyses while comparing serum parameters were age, gender, BMI, physical activity, smoking, and alcohol drinking. For cognitive analysis, factors including age, gender, BMI, education, and physical activity were adjusted. Chi-square test was used for binary categorical variables difference between groups. Multiple logistic regression analysis was applied to identify the factors that affect the incidence of MCI using the stepwise variable selection method. Statistical significance was set at *P* < 0.05.

## Results

### Demographic Characteristics of the Participants

The demographic characteristics of all the participants were described in Table 1. A total of 500 adults participated in the study, while 28 subjects were excluded due to their incapability of completing the questionnaires, unsuccessful ApoE genotyping or serum parameters measurement. A total of 472 individuals were finally included in the subsequent analysis. The average BMI of the participants was 25.81 ± 8.90. MCI subjects were significantly older than control subjects. The mean age of control and MCI participants was 62.09 ± 5.73 and 63.87 ± 6.9 years, respectively (*P* < 0.01). After categorizing the subjects according to age, we further found that, with the increase of age, the proportion of MCI increased gradually; and the highest proportion of MCI was observed in the age of 70 + group (*P* < 0.05) (Table A1 in Appendix). The proportion of subjects with smoking habit was higher in MCI group than observed in control group (*P* < 0.05). Furthermore, the MCI subjects had significantly less physical activity than control subjects (*P* < 0.01). They also demonstrated higher serum TC and HDL-C status than control subjects (*P* < 0.01).

**Table 1:** Demographic characteristic of the participants.

Demographic character	Cognition		Total (*n* = 472)	*P*-value
	Normal (*n* = 328)	MCI (*n* = 144)		
Age (year), mean ± SD	62.09 ± 5.73	63.87 ± 6.9	62.63 ± 6.16	0.007
Age distribution, ***n*** (%) (year)				
50–60	159 (48.5)	56 (38.9)	215 (36.0)	0.017
60–70	142 (43.3)	64 (44.4)	206 (50.4)	
>70	27 (8.2)	24 (16.7)	51 (13.6)	
BMI (kg/m^2^), mean ± SD	25.84 ± 10.42	25.73 ± 4.17	25.81 ± 8.90	0.904
Gender, *n* (%)				0.035
Male	84 (25.6)	51 (35.4)	135 (28.6)	
Female	244 (74.3)	93 (64.5)	337 (71.4)	
Education, *n* (%)				0.003
Illiterate	20 (6.1)	8 (5.6)	28 (5.9)	
Primary school	71 (21.6)	32 (22.2)	103 (21.8)	
Junior high school	130 (39.6)	80 (55.6)	210 (44.5)	
High school	86 (26.2)	23 (16.0)	109 (23.1)	
Junior college	13 (4.0)	1 (0.7)	14 (3.0)	
Undergraduate and above	8 (2.4)	0 (0.0)	8 (1.7)	
Smoking, *n* (yes, %)	39 (11.9)	29 (20.1)	68 (14.4)	0.023
Alcohol drinking, *n* (yes, %)	90 (27.4)	38 (26.4)	128 (27.1)	0.910
Physical activity, *n* (%)				0.003
Never	20 (6.1)	23 (16.0)	43 (9.1)	
1–3 times/week	45 (13.7)	17 (11.8)	62 (13.1)	
4–5 times/week	38 (11.6)	8 (5.6)	46 (9.7)	
Everyday	225 (68.6)	96 (66.7)	321 (68.0)	
ApoE genotype, *n* (%)				0.074
E2	48 (14.6)	31 (21.5)	79 (16.7)	
E3	231 (70.4)	87 (60.4)	318 (67.4)	
E4	49 (14.9)	26 (18.1)	75 (15.9)	
Serum parameters, mean ± SE				
GLU (mmol/L)	6.29 ± 1.86	6.37 ± 1.60	6.31 ± 0.08	0.682
TC (mmol/L)	5.19 ± 1.13	5.43 ± 1.16	5.26 ± 0.05	0.009
TG (mmol/L)	2.23 ± 1.79	1.89 ± 1.12	2.13 ± 0.08	0.061
LDL-C (mmol/L)	3.01 ± 0.89	3.00 ± 0.84	3.01 ± 0.04	0.735
HDL-C (mmol/L)	1.39 ± 0.28	1.48 ± 0.30	1.42 ± 0.01	0.000
Estradiol (pg/ml)	18.86 ± 13.92	22.45 ± 13.65	19.95 ± 0.64	0.099
Cortisol (ng/ml)	210.41 ± 49.85	210.69 ± 54.95	210.50 ± 2.37	0.841

*Chi-square test was used for binary categorical variables difference between groups. General linear model (GLM) method was used for the comparison of serum parameters. During the comparison of serum GLU, TC, TG, LDL-C, and HDL-C, confounding factors including age, sex, BMI, physical activity, smoking, and alcohol drinking were adjusted. During the comparison of serum estradiol and cortisol levels, confounding factors including age, sex, BMI, and physical activity were adjusted. *P*-value < 0.05 was considered as significance*.

*BMI, body mass index; ApoE, apolipoprotein E. TC, total cholesterol; TG, triglyceride; LDL-C, low-density lipoprotein cholesterol; HDL-C, high-density lipoprotein cholesterol*.

### Serum Parameters and Cognition According to ApoE Genotype

The ApoE genotype difference of serum parameters and cognition were compared in Table 2. Compared to ApoE3 subjects, ApoE2 and ApoE4 carriers significantly demonstrated a relatively higher serum TG concentration (*P* < 0.01). Moreover, highest mean serum HDL-C level was also detected in ApoE2 carriers (*P* < 0.05). ApoE4 carriers demonstrated the lowest mean serum HDL-C levels (*P* < 0.05). They also have a lower naming ability compared to ApoE3 and ApoE2 carriers (*P* < 0.05).

**Table 2:** Serum parameters and cognition according to ApoE genotype.

Parameters and cognition	ApoE genotype (*n* = 472)			*P*-value
	E2 (*n* = 79)	E3 (*n* = 318)	E4 (*n* = 75)	
**Parameters**				
GLU (mmol/l)	6.03 (5.62, 6.45)	6.41 (6.21, 6.61)	6.22 (5.81, 6.64)	0.415
TC (mmol/l)	5.34 (5.08, 5.60)	5.24 (5.12, 5.36)	5.27 (5.02, 5.53)	0.802
TG (mmol/l)	2.69 (2.31, 3.07)*	1.98 (1.80, 2.16)	2.20 (1.82, 2.58)	0.009
LDL-C (mmol/l)	2.79 (2.60, 2.99)*	3.04 (2.94, 3.14)	3.08 (2.88, 3.28)	0.074
HDL-C (mmol/l)	1.50 (1.44, 1.57)*	1.41 (1.38, 1.44)	1.37 (1.31, 1.44)**	0.024
Estradiol (pg/ml)	20.44 (17.64, 23.23)	20.14 (18.81, 21.48)	18.55 (15.82, 21.28)	0.546
Cortisol (ng/ml)	206.78 (194.86, 218.70)	212.76 (207.06, 218.46)	204.80 (193.15, 216.45)	0.386
**Cognition**				
MoCA score	22.69 (21.41, 23.96)	23.87 (23.26, 24.48)	22.54 (21.28, 23.80)	0.143
Visual and executive	3.38 (3.08, 3.67)	3.69 (3.55, 3.83)	3.45 (3.16, 3.74)	0.190
Naming	2.85 (2.72, 2.97)	2.90 (2.84, 2.96)	2.69 (2.57, 2.81)*	0.025
Attention	5.06 (4.75, 5.37)	5.17 (5.02, 5.31)	4.98 (4.68, 5.29)	0.738
Language	1.91 (1.71, 2.11)	2.12 (2.03, 2.22)	1.94 (1.74, 2.13)	0.101
Abstraction	1.35 (1.16, 1.54)	1.54 (1.45, 1.64)	1.41 (1.22, 1.60)	0.232
Memory and delayed recall	2.47 (2.13, 2.81)	2.72 (2.56, 2.89)	2.60 (2.27, 2.94)	0.426
Orientation	5.68 (5.42, 5.93)	5.74 (5.62, 5.86)	5.47 (5.22, 5.72)	0.299

*Data were expressed as mean (95% CI). General linear model (GLM) was used for data analysis. When comparing serum parameters, factors including gender, age, BMI, smoking, alcohol drinking, and physical activity were adjusted. When comparing cognition, factors including gender, age, BMI, smoking, alcohol drinking, physical activity, and education were adjusted. *P*-value < 0.05 was considered as significance*.

*Comparing with ApoE3 carriers, P < 0.05.

**Comparing with ApoE2 carriers, P < 0.05.

*GLU, glucose; TC, total cholesterol; TG, triglyceride; LDL-C, low-density lipoprotein cholesterol; HDL-C, high-density lipoprotein cholesterol; ApoE, apolipoprotein E*.

### Association of Serum Steroid Hormone Levels with Cognition

We categorized the participants according to the quartile of serum estrogen and cortisol levels into four groups (Q1-Q4). As shown in Table 3, subjects in the top quartile of serum estradiol (Q4) group have the lowest memory and delayed recall ability compared to the subjects in Q1 and Q2 groups (*P* < 0.05). As shown in Table 4, there was no significant difference between cognitive performances by serum cortisol status in the elderly participants (*P* > 0.05).

**Table 3:** Cognition according to serum estradiol level.

Cognition	Estradiol (μg/ml)				*P*-value
	Q1 (*n* = 118)	Q2 (*n* = 118)	Q3 (*n* = 118)	Q4 (*n* = 118)	
MoCA score	23.90 (22.89, 24.90)	23.94 (22.96, 24.91)	22.99 (22.03, 23.96)	22.91 (21.83, 23.99)	0.360
Visual–spatial and executive	3.61 (3.78, 3.83)	3.72 (3.50, 3.94)	3.56 (3.34, 3.78)	3.51 (3.27, 3.76)	0.642
Naming	2.89 (2.79, 2.99)	2.88 (2.77, 2.98)	2.81 (2.71, 2.91)	2.83 (2.72, 2.95)	0.654
Attention	5.20 (4.94, 5.45)	5.12 (4.88, 5.37)	5.02 (4.78, 5.26)	5.10 (4.83, 5.37)	0.790
Language	2.11 (1.95, 2.27)	2.09 (1.93, 2.24)	2.05 (1.90, 2.20)	1.98 (1.81, 2.15)	0.736
Abstraction	1.50 (1.34, 1.65)	1.62 (1.47, 1.77)	1.42 (1.28, 1.57)	1.41 (1.24, 1.57)	0.213
Memory and delayed recall	2.91 (2.64, 3.17)	2.79 (2.53, 3.05)	2.57 (2.31, 2.82)	2.38 (2.09, 2.66)*	0.037
Orientation	5.69 (5.48, 5.90)	5.71 (5.51, 5.92)	5.57 (5.36, 5.77)	5.72 (5.49, 5.95)	0.697

*Data were expressed as mean (95% CI). General linear model (GLM) was used for data analysis. Possible confounding factors including gender, age, BMI, smoking, physical activity, and education were adjusted. *P* < 0.05 was considered to be statistically significant*.

*Comparing with Q1 group, P < 0.05.

*MoCA, Montreal Cognitive Assessment; Q, quartile*.

**Table 4:** Cognition according to serum cortisol level.

Cognition	Cortisol (μg/ml)				*P*-value
	Q1 (*n* = 118)	Q2 (*n* = 118)	Q3 (*n* = 118)	Q4 (*n* = 118)	
MoCA score	22.92 (21.94, 23.89)	23.61 (22.65, 24.58)	23.73 (22.76, 24.70)	23.46 (22.49, 24.43)	0.662
Visual–spatial and executive	3.54 (3.32, 3.76)	3.68 (3.46, 3.89)	3.52 (3.31, 3.74)	3.66 (3.44, 3.88)	0.696
Naming	2.87 (2.77, 2.97)	2.83 (2.73, 2.93)	2.83 (2.73, 2.93)	2.88 (2.78, 2.99)	0.826
Attention	4.89 (4.65, 5.13)	5.13 (4.89, 5.37)	5.23 (4.99, 5.47)	5.18 (4.93, 5.42)	0.217
Language	2.06 (1.91, 2.21)	2.09 (1.94, 2.24)	2.05 (1.89, 2.20)	2.03 (1.88, 2.18)	0.966
Abstraction	1.40 (1.25, 1.55)	1.52 (1.37, 1.66)	1.49 (1.34, 1.64)	1.55 (1.40, 1.70)	0.529
Memory and delayed recall	2.57 (2.31, 2.82)	2.76 (2.51, 3.02)	2.87 (2.61, 3.13)	2.44 (2.18, 2.70)	0.088
Orientation	5.60 (5.40, 5.81)	5.61 (5.41, 5.81)	5.74 (5.54, 5.95)	5.73 (5.52, 5.93)	0.674

*Data were expressed as mean (95% CI). General linear model (GLM) was used for data analysis. Possible confounding factors including gender, age, BMI, smoking, physical activity, and education were adjusted. *P* < 0.05 was considered to be statistically significant*.

*MoCA, Montreal Cognitive Assessment; Q, quartile*.

### Cognition According to Serum Steroid Hormone Levels and ApoE Genotype

The association of serum estradiol status and cognitive performance by ApoE genotype were observed (Table 5). ApoE2 carriers with the highest serum estradiol status (Q4) have the lowest visual-spatial and executive ability (*P* < 0.05). Compared to ApoE3 and E2 subjects in Q1 group, ApoE4 carriers have the highest naming abilities, while the E4 subject in Q3 group have the lowest naming ability (*P* < 0.05). Furthermore, difference of visual–spatial and executive ability and naming ability across estradiol quartiles by ApoE genotype were also demonstrated (*P* < 0.05).

**Table 5:** Cognition according to serum estradiol level and ApoE genotype.

Cognition	ApoE genotype	Estradiol (μg/ml)				*P*-value
		Q1 (*n* = 118)	Q2 (*n* = 118)	Q3 (*n* = 118)	Q4 (*n* = 118)	
MoCA score	E2 (*n* = 79)	24.40 (22.15, 26.65)	23.34 (21.12, 25.55)	21.85 (18.97, 24.73)	21.21 (18.55, 23.86)	0.600
	E3 (*n* = 318)	23.81 (22.58, 25.04)	24.47 (23.31, 25.64)	23.71 (22.56, 24.87)	23.47 (22.21, 24.73)^b^	
	E4 (*n* = 75)	23.83 (21.38, 26.28)	21.87 (19.10, 24.64)	20.98 (18.82, 23.14)	21.85 (19.54, 24.16)	

Visual–spatial and executive	E2 (*n* = 79)	3.89 (3.39, 4.40)	3.66 (3.16, 4.16)	3.16 (2.51, 3.81)	2.75 (2.15, 3.35)*^,^**	0.028
	E3 (*n* = 318)	3.55 (3.27, 3.83)	3.84 (3.58, 4.10)	3.65 (3.39, 3.91)	3.67 (3.39, 3.96)^b^	
	E4 (*n* = 75)	3.55 (3.00, 4.10)	3.19 (2.56, 3.81)	3.46 (2.97, 3.95)	3.42 (2.89, 3.94)^b^	

Naming	E2 (*n* = 79)	2.92 (2.69, 3.15)	2.83 (2.61, 3.06)	2.88 (2.59, 3.18)	2.75 (2.47, 3.02)	0.041
	E3 (*n* = 318)	2.87 (2.74, 3.00)	2.94 (2.82, 3.06)^a^	2.92 (2.80, 3.04)^a^	2.88 (2.75, 3.01)	
	E4 (*n* = 75)	2.98 (2.73, 3.23)	2.58 (2.29, 2.87)	2.38 (2.16, 2.60)*	2.69 (2.45, 2.93)	

Attention	E2 (*n* = 79)	5.20 (4.63, 5.77)	5.09 (4.53, 5.65)	4.93 (4.20, 5.66)	5.14 (4.47, 5.81)	0.935
	E3 (*n* = 318)	5.22 (4.91, 5.53)	5.19 (4.89, 5.48)	5.16 (4.87, 5.45)	5.10 (4.78, 5.42)	
	E4 (*n* = 75)	5.12 (4.50, 5.74)	4.80 (4.10, 5.50)	4.57 (4.02, 5.11)	5.02 (4.43, 5.60)	

Language	E2 (*n* = 79)	2.18 (1.82, 2.53)	1.86 (1.51, 2.21)	1.69 (1.24, 2.14)	1.98 (1.56, 2.39)	0.4468
	E3 (*n* = 318)	2.11 (1.91, 2.30)	2.15 (1.97, 2.34)^b^	2.19 (2.01, 2.37)^b^	2.02 (1.82, 2.22)	
	E4 (*n* = 75)	2.05 (1.67, 2.44)	2.05 (1.61, 2.48)	1.73 (1.39, 2.06)	1.80 (1.44, 2.16)	

Abstraction	E2 (*n* = 79)	1.42 (1.08, 1.77)	1.56 (1.22, 1.91)	1.18 (0.74, 1.63)	1.22 (0.81, 1.63)	0.830
	E3 (*n* = 318)	1.53 (1.34, 1.72)	1.71 (1.53, 1.89)^a^	1.47 (1.29, 1.65)**	1.45 (1.26, 1.65)	
	E4 (*n* = 75)	1.46 (1.08, 1.84)	1.24 (0.81, 1.67)	1.41 (1.07, 1.74)	1.35 (0.99, 1.71)	

Memory and delayed recall	E2 (*n* = 79)	3.05 (2.45, 3.65)	2.68 (2.09, 3.27)	2.33 (1.55, 3.10)	1.77 (1.06, 2.48)*	0.773
	E3 (*n* = 318)	2.92 (2.59, 3.25)	2.83 (2.52, 3.14)	2.63 (2.32, 2.94)	2.53 (2.20, 2.87)^b^	
	E4 (*n* = 75)	2.72 (2.07, 3.38)	2.79 (2.05, 3.53)	2.43 (1.85, 3.01)	2.20 (1.58, 2.82)	

Orientation	E2 (*n* = 79)	5.74 (5.26, 6.21)	5.65 (5.18, 6.12)	5.68 (5.07, 6.29)	5.63 (5.07, 6.19)	0.258
	E3 (*n* = 318)	5.62 (5.36, 5.88)	5.81 (5.57, 6.06)^a^	5.70 (5.45, 5.94)^a^	5.82 (5.56, 6.09)	
	E4 (*n* = 75)	5.94 (5.42, 6.45)	5.23 (4.65, 5.82)	5.01 (4.56, 5.47)	5.39 (4.90, 5.88)	

*Data were expressed as mean (95% CI). General linear model (GLM) was used for data analysis. Possible confounding factors including gender, age, BMI, smoking, physical activity, and education were adjusted. *P* < 0.05 was considered to be statistically significant*.

*Comparing with Q1 group, P < 0.05.

**Comparing with Q2 group, P < 0.05.

*^a^Comparing with E4 carriers, P < 0.05*.

*^b^Comparing with E2 carriers, P < 0.05*.

*ApoE, apolipoprotein E; MoCA, Montreal Cognitive Assessment; Q, quartile*.

As shown in Table 6, the association of serum cortisol with language ability by ApoE genotype was detected. Language ability of ApoE2 carriers diminished accordingly with the increase of serum cortisol status; and the lowest language ability was observed in ApoE2 carriers in Q4 group (*P* < 0.05). ApoE4 carriers with Q3 level of serum cortisol also demonstrated diminished language ability (*P* < 0.05). Additionally, difference of language ability across cortisol quartiles by ApoE genotype was indicated in the current study (*P* < 0.05).

**Table 6:** Cognition according to serum cortisol level and ApoE genotype.

Cognition	ApoE genotype	Cortisol (μg/ml)				*P*-value
		Q1 (*n* = 118)	Q2 (*n* = 118)	Q3 (*n* = 118)	Q4 (*n* = 118)	
MoCA score	E2 (*n* = 79)	23.29 (20.84, 25.74)	23.67 (21.07, 26.28)	22.87 (20.81, 24.92)	21.51 (18.61, 24.41)	0.733
	E3 (*n* = 318)	22.95 (21.79, 24.11)	24.14 (22.91, 25.38)	24.21 (23.02, 25.40)^b^	24.19 (23.08, 25.31)^b^	
	E4 (*n* = 75)	23.36 (19.82, 24.90)	22.26 (20.36, 24.17)	22.63 (19.84, 25.42)	20.86 (18.16, 23.57)	

Visual–spatial and executive	E2 (*n* = 79)	3.55 (3.00, 4.11)	3.49 (2.90, 4.08)	3.58 (3.11, 4.04)	2.94 (2.29, 3.60)	0.448
	E3 (*n* = 318)	3.57 (3.30, 3.83)	3.80 (3.52, 4.08)	3.52 (3.25, 3.79)	3.83 (3.58, 4.08)	
	E4 (*n* = 75)	3.44 (2.86, 4.01)	3.48 (3.05, 3.91)	3.46 (2.83, 4.09)	3.25 (2.64, 3.86)	

Naming	E2 (*n* = 79)	3.00 (2.74, 3.25)	2.96 (2.68, 3.23)	2.73 (2.52, 2.95)	2.73 (2.43, 3.03)	0.462
	E3 (*n* = 318)	2.86 (2.74, 2.98)	2.89 (2.77, 3.02)^a^	2.89 (2.77, 3.01)	2.96(2.84, 3.08)^a^	
	E4 (*n* = 75)	2.76 (2.50, 3.03)	2.60 (2.40, 2.80)	2.67 (2.38, 2.96)	2.58 (2.29, 2.86)	

Attention	E2 (*n* = 79)	4.92 (4.31, 5.54)	5.21 (4.56, 5.86)	5.24 (4.72, 5.75)	4.97 (4.25, 5.70)	0.711
	E3 (*n* = 318)	4.88 (4.59, 5.17)	5.19 (4.88, 5.50)	5.25 (4.96, 5.55)	5.33 (5.05, 5.61)^a^	
	E4 (*n* = 75)	4.88 (4.24, 5.52)	4.97 (4.49, 5.44)	5.07 (4.37, 5.77)	4.43 (3.76, 5.11)	

Language	E2 (*n* = 79)	2.13 (1.75, 2.51)	2.25 (1.84, 2.65)	1.78 (1.46, 2.10)**	1.61 (1.16, 2.07)**	0.039
	E3 (*n* = 318)	2.00 (1.82, 2.18)	2.15 (1.96, 2.34)^a^	2.20 (2.02, 2.39)^a^^,^^b^	2.13 (1.96, 2.30)^b^	
	E4 (*n* = 75)	2.26 (1.86, 2.65)	1.82 (1.53, 2.12)	1.66 (1.23, 2.10)	1.81 (1.39, 2.23)	

Abstraction	E2 (*n* = 79)	1.22 (0.84, 1.62)	1.60 (1.19, 2.00)*	1.35 (1.03, 1.67)*	1.37 (0.92, 1.82)*	0.969
	E3 (*n* = 318)	1.45 (1.27, 1.63)	1.56 (1.37, 1.75)	1.56 (1.38, 1.75)	1.60 (1.42, 1.77)	
	E4 (*n* = 75)	1.36 (0.97, 1.75)	1.38 (1.09, 1.68)	1.31 (0.88, 1.74)	1.43 (1.01, 1.85)	

Memory and delayed recall	E2 (*n* = 79)	2.75 (2.10, 3.40)	2.52 (1.82, 3.21)	2.45 (1.91, 3.00)	2.36 (1.59, 3.13)	0.769
	E3 (*n* = 318)	2.62 (2.31, 2.93)	2.81 (2.48, 3.14)	3.03 (2.71, 3.34)^b^	2.48 (2.19, 2.78)*^,^**	
	E4 (*n* = 75)	2.13 (1.45, 2.81)	2.73 (2.23, 3.24)	2.79 (2.05, 3.54)	2.26 (1.54, 2.98)	

Orientation	E2 (*n* = 79)	5.72 (5.20, 6.24)	5.65 (5.10, 6.20)	5.74 (5.31, 6.18)	5.53 (4.91, 6.14)	0.676
	E3 (*n* = 318)	5.59 (5.35, 5.84)	5.74 (5.48, 6.00)	5.76 (5.51, 6.01)	5.86 (5.63, 6.10)^a^	
	E4 (*n* = 75)	5.52 (4.99, 6.06)	5.28 (4.88, 5.68)	5.67 (5.08, 6.26)	5.11 (4.54, 5.68)	

*Data were expressed as mean (95% CI). General linear model (GLM) was used for data analysis. Possible confounding factors including gender, age, BMI, smoking, physical activity, and education were adjusted. *P* < 0.05 was considered to be statistically significant*.

*Comparing with Q1 group, P < 0.05.

**Comparing with Q2 group, P < 0.05.

*^a^Comparing with E4 carriers, P < 0.05*.

*^b^Comparing with E2 carriers, P < 0.05*.

*ApoE, apolipoprotein E; MoCA, Montreal Cognitive Assessment; Q, quartile*.

### Steroid Hormones and ApoE Genotype Affect the Occurrence of MCI

The association of serum steroids concentrations and ApoE genotype with the risk of MCI in the elderly was explored by using multiple logistic regression analysis after adjusting the potential confounding factors including age, BMI, gender, educational level, smoking, and physical activity level (Table 7). The MCI risk for the subjects with Q3 or Q4 quartile of serum estradiol level increased by 1.958 (95% CI: 1.107, 3.466; *P* = 0.021) and 2.004 (95% CI: 1.135, 3.540; *P* = 0.017), respectively. Interaction between serum estradiol concentration and ApoE genotype in affecting the risk of MCI was also observed. ApoE2 carriers with high serum estradiol (Q4 quartile) have increased risk of MCI than ApoE3 subjects with the low quartile (Q1) of serum estradiol level (*P* < 0.05). The interaction between ApoE genotype and serum cortisol level and the risk of MCI was only observed in the subjects with top quartile (Q4) of serum cortisol status. ApoE2 carriers with Q4 quartile of serum cortisol level demonstrated a potential increase in the risk for MCI (OR = 3.353; 95% CI: 1.135, 9.907; *P* = 0.029).

**Table 7:** Serum steroid, ApoE genotype, and the risk of MCI.

Item	*B*	SE	Wald	OR	95% CI	*P*-value
**Independent effects of estradiol**						
Q1 (reference)		–	9.256	1.000	–	0.026
Q2	0.139	0.305	0.208	1.149	0.633, 2.087	0.648
Q3	0.672	0.291	5.328	1.958	1.107, 3.466	0.021
Q4	0.695	0.219	5.735	2.004	1.135, 3.540	0.017

**Independent effects of cortisol**						
Q1 (reference)	–	–	2.228	1.000	–	0.526
Q2	−0.211	0.282	0.056	0.810	0.465, 1.408	0.454
Q3	−0.339	0.287	1.396	0.713	0.406, 1.250	0.237
Q4	0.025	0.276	0.008	1.026	0.597, 1.762	0.927

**Independent effects of ApoE genotype**						
E3 (reference)	–	–	4.543	1.000	–	0.103
E2	0.608	0.315	3.721	1.838	0.990, 3.410	0.054
E4	0.377	0.304	1.543	1.458	0.804, 2.644	0.214

**Synergistic effects of estradiol and ApoE genotype**						
Q1 × E3 (reference)	–	–	12.305	1.000	–	0.055
Q2 × E2	0.428	0.459	0.869	1.534	0.624, 3.768	0.351
Q2 × E4	0.071	0.603	0.014	1.073	0.329, 3.502	0.906
Q3 × E2	1.141	0.569	4.026	3.131	1.027, 9.546	0.045
Q3 × E4	0.900	0.434	4.304	2.460	1.051, 5.758	0.038
Q4 × E2	1.105	0.500	4.882	3.019	1.133, 8.046	0.027
Q4 × E4	0.294	0.478	0.379	1.342	0.526, 3.423	0.538

**Synergistic effects of cortisol and ApoE genotype**						
Q1 × E3 (reference)	–	–	7.604	1.000	–	0.269
Q2 × E2	−0.176	0.589	0.090	0.838	0.264, 2.660	0.765
Q2 × E4	0.517	0.391	1.748	1.677	0.779, 3.606	0.186
Q3 × E2	0.229	0.425	0.291	1.257	0.547, 2.891	0.590
Q3 × E4	0.635	0.553	1.318	1.886	0.638, 5.573	0.251
Q4 × E2	1.210	0.553	4.792	3.353	1.135, 9.907	0.029
Q4 × E4	−0.089	0.596	0.023	0.914	0.285, 2.939	0.881

*Logistic regression models were created to evaluate the independent and synergistic effects of serum steroids and/or ApoE genotype on the risk of MCI. Confounding factors such as age, sex, BMI, education, smoking, and physical activity levels were adjusted during analysis*.

*MCI, mild cognitive impairment; OR, odds ratio; CI, confidence interval; Q, quartile*.

## Discussion

The demographic characters of the participants were compared; and the differences in age, educational level and lifestyle between MCI and control subjects were observed. Our results were consistent with previously published studies (35–37). Comprehensively, these data demonstrate that age, educational level, physical activity, and smoking are influencing factors associated with cognitive performance in elderly Chinese. We did not observe any difference of ApoE genotype frequency between control and MCI subjects. The inconsistency of our study with other published studies may be attributed to the specifically studied population. In the current study, all the participants were non-demented community-dwellers, while, in other studies, the participants were clinically diagnosed cognitive impaired subjects or AD patients (38, 39).

Studies have indicated that an increase of serum cholesterol concentration is a potential risk factor for cognitive decline and dementia (40–43). In this current study, MCI subjects demonstrated relatively higher serum TC levels than normal subjects. However, a 7-year follow-up study (42) and the Framingham cohort study (44) did not observe any relationship between serum TC levels and the risk of AD (45). Siest and Merched also reported that AD patients have lower serum TC and HDL-C concentration than controls (46, 47). An inverse relationship between serum TC levels and the risk of dementia observed in an aged population (70 years old and above) (48) indicate that age dependent changes affecting serum TC levels and the time for serum TC measurements in different studies might contribute to these conflicting results.

After grouping the subjects according to ApoE genotypes, we found that serum lipid profile was ApoE genotype associated. ApoE genotype-dependent cognitive performance was also observed, demonstrated by the diminished naming ability in ApoE4 carriers compared to ApoE2 and ApoE3 carriers. As a receptor-binding ligand, ApoE is mainly known to be responsible for the transportation and metabolism of very LDL and HDL, and thus involved in the regulation of serum lipid profile (49). Furthermore, ApoE genetic variation-associated lipid metabolism disorder was suggested to be involved in the pathological progression of AD (50). Although the gene-dose effect of ApoE ε4 allele on the risk of both cognitive decline and AD has been indicated by previous studies (51–53), to date, the ApoE ε4 allele is solely suggested as a risk factor for AD rather than its cause (54, 55). All these results indicate that the correlation between ApoE genotype and cognitive performance in the elderly population might be potentially attributed to the concurring disturbance of lipid metabolism.

Increasing laboratory- and animal-based studies undoubtedly prove the neuroprotective effect(s) of estrogen. Lebrun’s population-based study also demonstrated a negative correlation between circulating estrogen content with the risk of MCI (56). However, contradictory outcomes were concluded from clinical use of estrogen for the treatment and prevention of AD. Yaffe’s study indicated that a high circulating estrogen levels predict a high risk of cognitive decline (57). Also, higher midlife circulating estrogen in male subjects predicted a smaller left and right brain occipital lobe volumes in late life (58). A cohort study found that higher circulating estrogen concentrations corresponded with a decline in verbal fluency in postmenopausal women (59). In the current study, we found a decreased tendency of memory and delayed recall ability accompanied with the increase of serum estradiol levels in the elderly. This result was in agreement with Johansson’s case-control study, in which the author detected an increase of serum estrogen concentration in both male and female MCI patients (60).

Physiologic relationship between estrogen and apolipoprotein has also been well established (61). The involvement of ApoE in the metabolism of cholesterol indicates the possible interaction between ApoE genetic polymorphism with cholesterol-derived steroids in affecting cognition in the elderly population. In ApoE4 negative women, estrogen replacement therapy (ERT) could reduce the risk of AD by 80%; but in female ApoE4 carriers, ERT could not affect their susceptibility to AD (62). These findings suggest that the mechanism(s) involved and efficacy of *in vivo* estrogen and/or ERT in the prevention of AD was ApoE genotype dependent. Our data further consent to the involvement of ApoE genotype in the relationship between *in vivo* estradiol levels and cognitive performance in aging subjects. The visual-spatial and executive function of ApoE2 carriers and naming ability of ApoE4 carriers were correlated with changes in serum estrogen concentration. Moreover, the interaction of serum estradiol and ApoE genotype on the risk of MCI in this older Chinese population was also indicated by the significantly increased risk of MCI in ApoE2 carriers with Q3 or Q4 quartile level of serum estradiol status. ApoE2 has been suggested as having a protective effect against cognitive decline and delaying the aging of brain in AD (63). However, the AD preventing effect of ApoE2 is generally observed in E2/E3 individuals or in E2 homozygous females (64, 65). The relationship between ApoE2 and AD-like neuropathologic features appear to be very complex. ApoE2 has also been reported to increase Alzheimer pathology (specifically neuritic plaques) in the elderly (66). Study of Swedish individuals found that ApoE2 was associated with a reduced risk of AD only in females (67). The carriage of the ApoE2 is associated with a greater risk for hyperinsulinemia and diabetes, both of which increase the risk for AD (68, 69). The synergistic effects of estrogen and ApoE in affecting multiple pathogenic mechanisms of AD have been reported (70). Nathan et al. reported an ApoE genotype-dependency on neurite outgrowth effect with estrogen status (71). It was also found that estrogen could activate glucose metabolism system and suppress ketogenic system in ApoE 2 carriers (72). Therefore, in ApoE2 carriers, high estradiol status would result in higher glucose metabolism and suppression of the ketogenic system in brain, and put brain at metabolic risk (73). All these data indicated a complex interaction between ApoE genotype, estradiol and brain function. Although, up to date, only few studies have explored the interaction of ApoE2 with sex steroids in affecting cognition, outcomes from ours and others’ studies demonstrate an ApoE genotype dependent association of *in vivo* estrogen status and cognitive function. The association between ApoE2 and estradiol remains yet to be fully clarified.

In fact, less evidence has been contributed to the association of cortisol levels with cognition. It has been proposed that memory performance could be modulated by circulating cortisol in an “inverted-U shape” way (74–76). In contrast to these findings, several studies have shown that AD patients have higher basal circulating cortisol concentration than healthy control subjects (77). Chronically elevated circulating cortisol level initiated by the dysregulation of the HPA axis was often associated with accelerated declivity in cognitive functioning of the elderly (78–80). Lind et al. study observed an increased awakening salivary cortisol levels in MCI subjects compared to elderly controls (81). In the current study, we did not observe serum cortisol dependent differences in cognitive performance in these elderly subjects. Likewise, logistic analysis results did not also indicate any significance between serum cortisol with the risk of MCI in the elderly Chinese subjects. However, after taking ApoE genotype into account, it is interesting to observe that ApoE2 and ApoE4 carriers with relatively higher serum cortisol levels demonstrated lower language ability; and the genotype–cortisol interaction in affecting language ability was significant. Further logistic analysis also found an interaction of ApoE2 genotype and higher serum cortisol with the risk of MCI. More recently, a dose-dependent effect of ApoE4 and ApoE2 genotypes on behavioral symptoms in patients with frontotemporal dementia was indicated by a prospective study (82). Other studies have also reported a relationship between cortisol status, changes in regional brain volumes and cognitive function. For example, high circulating cortisol status was found to be correlating with brain atrophy especially in bilateral hippocampal and cerebellar gray matter (83). Hydrocortisone challenge was also found to dramatically decrease hippocampal volume in healthy subjects (84). Together with our present results, all these data are in line with the hypothesis that ApoE genotype and circulating cortisol potentially contribute to cognitive performance. The higher circulating cortisol in ApoE2 or E4 carriers may indicate a greater risk for cognitive decline.

Several limitations need to be considered in this present study. All the participants were community residents from the Beijing city. This implies that the extrapolation of our conclusions with regard to other populations should be carefully noted. Furthermore, the cross-sectional design of the present study renders weakness. We could not follow-up on the prandial changes of sex steroids *in vivo*. Finally, although, all factors known to be associated with cognition or serum parameter levels were adjusted, some other unidentified factors may have influence on the results and consequently on the overall ApoE genotype-dependent cognitive outcomes.

## Conclusion

The current study explored the association between serum steroids and cognitive function in non-demented older Chinese subjects with different ApoE genotypes. Our data indicate an association of circulating steroid hormone with cognition. Also the relationship between steroid hormones and cognitive performance in non-demented older Chinese subjects was ApoE-genotype associated. ApoE2 carriers with higher serum steroids might be predictive of having an increased risk of developing MCI.

## Ethics Statement

This study was carried out in accordance with the recommendations of “The Medical Ethics Committee of Capital Medical University (No. 2012SY23)” with written informed consent from all subjects. All subjects gave written informed consent in accordance with the Declaration of Helsinki. The protocol was approved by the “The Medical Ethics Committee of Capital Medical University.”

## Author Contributions

LY gave contribution to the design of the work and the writing of the article. XH, JZ, and TL did the contributed to the biomarkers measurements. SD, HZ, YZ, and JZ contributed to the cognitive function test. NVHL, SD, and LY contributed to the analysis and interpretation of data. LY, XH, TL, SD, HZ, JZ, YZ, and NVHL gave the approval of the final version to be published.

## Conflict of Interest Statement

The authors declare that the research was conducted in the absence of any commercial or financial relationships that could be construed as a potential conflict of interest.

## Appendix

**Table A1:** MCI, ApoE genotype, and serum parameters according to age.

Items	Age (years)			*P* value
	50–60 (*n* = 215)	60–70 (*n* = 206)	>70 (*n* = 51)	
MCI, *n* (%)	56 (26.0)	64 (31.1)	24 (47.1)	0.017
ApoE genotype, *n* (%)				0.419
E2	31 (14.4)	36 (17.5)	12 (23.5)	
E3	148 (68.8)	138 (67.0)	32 (62.7)	
E4	36 (16.7)	32 (15.5)	7 (13.7)	

**Serum parameters (mean ± SE)**				
GLU (mmol/l)	6.37 (6.13, 6.61)	6.19 (5.94, 6.44)	6.59 (6.09, 7.08)	0.320
TC (mmol/l)	5.34 (5.19, 5.49)	5.14 (4.98, 5.29)	5.39 (5.09, 5.70)	0.117
TG (mmol/l)	2.19 (1.97, 2.41)	2.09 (1.86, 2.32)	1.97 (1.52, 2.42)	0.635
HDL-C (mmol/l)	1.41 (1.37, 1.45)	1.40 (1.37, 1.44)	1.49 (1.41, 1.57)	0.132
LDL-C (mmol/l)	3.07 (2.95, 3.18)	2.93 (2.82, 3.05)	3.00 (2.77, 3.24)	0.282
Estradiol (pg/ml)	19.83 (18.16, 21.51)	19.80 (18.07, 21.54)	20.57 (17.12, 24.02)	0.922
Cortisol (ng/ml)	208.75 (201.73, 215.77)	211.62 (204.34, 218.90)	211.07 (196.60, 225.55)	0.850

*MCI, mild cognitive impairment; ApoE, Apolipoprotein E; TC, total cholesterol; TG, triglyceride; LDL-C, low density lipoprotein cholesterol; HDL-C, high density lipoprotein cholesterol*.

*Chi-square test was used for binary categorical variables difference between groups. General Linear Model method was used for the comparison of serum parameters. During the comparison of serum lipids status, confounding factors including sex, BMI, physical activity, smoking, and alcohol drinking were adjusted. During the comparison of serum estradiol and cortisol levels, confounding factors including sex, BMI, physical activity were adjusted. P value <0.05 was considered as significance*.
